# Mathematical Models of Tuberculosis Reactivation and Relapse

**DOI:** 10.3389/fmicb.2016.00669

**Published:** 2016-05-17

**Authors:** Robert S. Wallis

**Affiliations:** The Aurum InstituteJohannesburg, South Africa

**Keywords:** tuberculosis, mathematical model, relapse, reactivation

## Abstract

The natural history of human infection with *Mycobacterium tuberculosis (Mtb)* is highly variable, as is the response to treatment of active tuberculosis. There is presently no direct means to identify individuals in whom *Mtb* infection has been eradicated, whether by a bactericidal immune response or sterilizing antimicrobial chemotherapy. Mathematical models can assist in such circumstances by measuring or predicting events that cannot be directly observed. The 3 models discussed in this review illustrate instances in which mathematical models were used to identify individuals with innate resistance to *Mtb* infection, determine the etiologic mechanism of tuberculosis in patients treated with tumor necrosis factor blockers, and predict the risk of relapse in persons undergoing tuberculosis treatment. These examples illustrate the power of various types of mathematic models to increase knowledge and thereby inform interventions in the present global tuberculosis epidemic.

## Introduction

The natural history of human infection with *Mycobacterium tuberculosis (Mtb)* is highly variable. Some individuals appear able to eradicate the infection spontaneously, either with or without expansion of *Mtb*-specific T cells. In others, the infection is contained but not eradicated, resulting in a latent *Mtb* infection (LTBI) that may reactivate years or even decades later. In the most highly susceptible individuals, *Mtb* infection may progress directly to active disease, without an intervening period of latency.

The response to tuberculosis treatment is similarly variable, due to the recurrence of active disease in some patients ostensibly appearing cured at the end of treatment. There is presently no direct means to identify individuals in whom *Mtb* infection has been eradicated, whether by a bactericidal immune response or sterilizing antimicrobial chemotherapy. Indeed, LTBI presently can only be definitively identified by its capacity to reactivate or relapse. This inability has hindered basic research and has delayed tuberculosis vaccine and drug development.

Mathematical models can assist in such circumstances by measuring or predicting events that cannot be directly observed. Markov models describe time-dependent transitions among states of a system (Bhat and Miller, 2002), such as the acquisition of *Mtb* infection and progression to active tuberculosis. Hidden Markov models can reveal transitions that cannot be observed directly, such as those preceding active tuberculosis, by the analysis of fluxes through observable transitions. This approach, which is commonly used in voice recognition software and DNA sequencing, had not previously been applied in tuberculosis. Mathematical models can also predict clinical events that have not yet occurred, based on analysis of markers associated with the long-term outcome of interest. Both approaches can use aggregated data to assess events in cohorts when individual outcomes cannot be ascertained.

Three models will be discussed in this review. In the first, which has not previously been published, a Markov model was used to assess the likelihood of enhanced innate resistance to *Mtb* infection in individuals remaining tuberculin skin test (TST) negative despite repeated apparent exposure. In the second, a hidden Markov model was used to determine the relative involvements of reactivation of latent infection vs. progression of new infection to the development of active tuberculosis in persons treated with tumor necrosis factor (TNF) antagonists (Wallis, 2008). Details from both models have been included to illustrate how they were created and solved using simple mathematical tools in Excel. In the last, a statistical model was developed to predict tuberculosis relapse risk based on treatment duration and proportion of individuals sputum culture positive after 2 months (Wallis et al., 2013, 2015). These examples illustrate the power of various types of mathematic models to increase knowledge and thereby inform interventions in the present global tuberculosis epidemic.

## Identifying innate resistance to *Mtb* infection

Transmission of *Mtb* infection occurs by inhalation of infected cough generated aerosols by active tuberculosis cases. As a result, the annual risk of acquiring *Mtb* infection (ARTI) is closely linked to tuberculosis prevalence. However, even in a high-burden country such as South Africa where the majority of adults are TST positive, TST negative status may indicate innate resistance to *Mtb* infection or merely lack of exposure. South African gold miners, however, are a unique subpopulation with extraordinarily high tuberculosis risk (3%/year in 2011; Churchyard et al., 2014). Molecular strain typing indicates at least 32% of cases in this population represent recent transmission (Mathema et al., 2015). Congregate working, living, and social conditions in the mines, combined with high prevalence rates of HIV and silicosis contribute to high levels of ongoing transmission and disease. Mathematical modeling has estimated the ARTI among miners is at least 20% (Vynnycky et al., 2015), roughly 5 times that in high-burden non-mining South African townships (Wood et al., 2010). However, despite this high level of *Mtb* exposure, one survey found 13% of 115 HIV-uninfected miners were uninfected (TST = 0 mm; Hanifa et al., 2009). The finding that this relatively large minority of individuals in this heavily exposed population remain uninfected was unanticipated.

**Figure 1:**
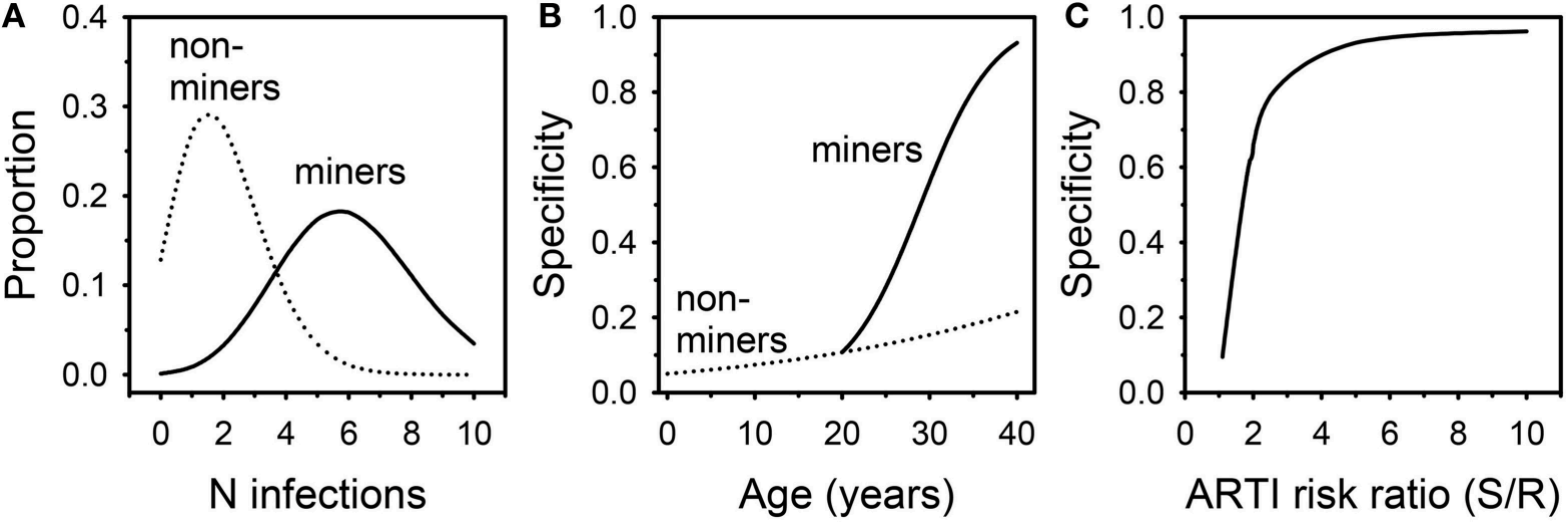
**Results of Markov modeling of *Mtb* infection in South African miners and non-miners**. **(A)** distribution of likely numbers of infection episodes in 40 year old miners and non-miners. **(B)** Specificity of negative TST for innate *Mtb* resistance in relation to age. **(C)** Specificity of negative TST for innate *Mtb* resistance in relation to degree of conferred resistance.

A Markov model was therefore developed to determine the likelihood that this unique population represented innate resistance to *Mtb* infection. It was initially assumed: (1) the ARTI in the susceptible South African non-mining population was 0.05; (2) this risk was increased 5x among susceptible miners; (3) work in the mines commenced at age 20 and was continuous through age 40; (4) the proportion of individuals with innate resistance to *Mtb* infection was 0.05 in both the mining and non-mining populations; and (5) individuals with the resistant phenotype had 1/5^*th*^ the ARTI risk of susceptible individuals. The model did not assess whether TST positive individuals could resist subsequent infections, nor did it account for removal of subjects due to active tuberculosis. An Excel file describing the model is provided as an online Supplementary Material. The model was then interrogated annually to examine *Mtb* infection status in miners and non-miners from birth to age 40 (Figure 1). The most likely number of *Mtb* infection episodes in susceptible 40 year olds increased from 2 in non-miners to 6 in miners (Figure 1A). The proportion of uninfected susceptible individuals dropped from 13% in non-miners to 0.1% in miners. As a result, the specificity of TST = 0 mm as an indicator of innate *Mtb* resistance improved from 22% in 40 year old non-miners to 93% in miners (Figure 1B). Specificity remained adequate until the degree of conferred resistance dropped below 2 (Figure 1C). The key finding from the modeling exercise was that, among individuals with a long history of employment underground in the mines in South Africa, negative TST status was highly likely to reflect innate resistance to *Mtb* infection. Interferon gamma release assay testing can help confirm the uninfected status of these individuals. Understanding the mechanism(s) of resistance may lead to therapeutic strategies targeting the host to counter immune evasion by *Mtb*.

## Determining the etiology of tuberculosis in patients receiving TNF blockers

TNF is required for host defenses against mycobacterial infection (Kindler et al., 1989). It is also central to the pathogenesis of inflammatory conditions such as rheumatoid arthritis (RA) and Crohn's disease. The 2 main classes of TNF blockers include antibody (e.g., infliximab) and soluble receptor (etanercept being the sole example at present). The 2 classes are considered therapeutically equivalent for RA. However, differences in function among these agents have emerged that appear to reflect differences in their structures. Infliximab and adalimumab, for example, are effective in the treatment of granulomatous conditions such as Crohn's disease and sarcoidosis, whereas etanercept is not (Sandborn et al., 2001; Hanauer et al., 2002; Utz et al., 2003). *In vitro*, TNF antibodies inhibit T cell activation and interferon gamma production, whereas etanercept does not (Saliu et al., 2006; Haider et al., 2007). In mice, both TNF antibody and its soluble receptor markedly increase mortality in acute *Mtb* infection, but only the antibody exacerbates chronic infection (Plessner et al., 2007), which is thought to model human latent infection. The basis of these observations is not fully understood.

**Figure 2:**
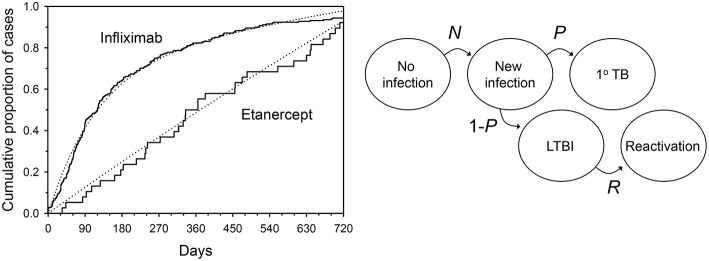
**Tuberculosis occurring during tumor necrosis factor blockade**. **Left**: Solid lines indicate time to onset distributions as reported to US FDA from January 1998 through March 2003. Dotted lines indicate the distributions resulting from a typical mathematical model during Monte Carlo simulation. **Right**: Hidden Markov model of tuberculosis etiology during TNF blockade. *N* = monthly proportion acquiring new infection with *Mycobacterium tuberculosis*; *P* = proportion of new infections progressing directly to active TB; *R* = monthly proportion of latent TB infections undergoing reactivation. From (Wallis, 2008), with permission.

Treatment with anti-TNF agents for conditions such as RA places patients at increased risk of tuberculosis (Keane et al., 2001; Wallis et al., 2004a,b). The time to tuberculosis onset after starting an anti-TNF agent provides an important clue from which its etiology can be discerned, since the clustering of cases shortly after the start of anti-TNF treatment is consistent with reactivation of latent infection. For example, the median time to tuberculosis onset after starting infliximab treatment is uniformly short (12–21 weeks) in all series in which this has been examined (Keane et al., 2001; Wallis et al., 2004a; Brassard et al., 2006). In contrast, this interval for etanercept is 3–5 times longer, nearly equaling the midpoint of the period of data collection in each study (solid lines, Figure 2 **left panel**, *P* < 0.001 by Kaplan-Meier log rank analysis). This pattern, in which cases accumulate linearly, could be consistent with progression of new infection to active disease as such events would occur at random during the period of observation. Alternatively, it could be due to inefficient reactivation of latent infection, or a combination of both mechanisms. These mechanisms cannot be readily distinguished by ordinary clinical observation.

In 2008, a hidden Markov model with 5 states was used to examine this phenomenon (Figure 2 **right panel**; Wallis, 2008). The model assumed: (1) no cases of active tuberculosis are present at baseline; (2) an unknown proportion of persons have LTBI at baseline (*L*); (3) the variables describing state transitions are *N* (the incidence of new infections each month); *R* (the proportion of latent infections that reactivate each month); and *P*, the proportion of new infections that progress directly to tuberculosis) are fixed and unknown. The two treatments were permitted to affect *P* and *R*, but not *L* or *N*. Predicted results were compared to those reported to the US FDA Adverse Events Reporting System by physicians caring for US and EU patients treated with anti-TNF agents (solid lines, Figure 2 **left panel**). An accuracy score was calculated as the mean squared difference between observed and predicted recurrences. The Solver module of Excel was used to iteratively determine values *L, N, P*, and *R* that minimized this score, using a generalized reduced gradient algorithm (Walsh and Diamond, 1995). The model was interrogated at monthly intervals to determine the number of tuberculosis cases. A range of case rate ratios for infliximab and etanercept spanning the reported values were then tested in a Monte Carlo simulation of 600 cases.

The results of a typical simulation are illustrated by the dotted lines in Figure 2. The close approximation of the observed and modeled time-to-onset distributions indicate a good fit of this model. A summary of the Markov model parameters derived from the Monte Carlo simulations is shown in Table 1. Although there was a 12.1-fold difference in the apparent monthly rate of reactivation of latent infection between the 2 treatments (*P* < 0.001 by Wilcoxon's signed rank test), they did not differ in their effects on new infection, in that both drugs resulted in progression of all new infections to active disease in all of the models tested. Both these findings are consistent with studies in mice (Plessner et al., 2007). Moreover, the apparent risk of new *Mtb* infection in this analysis, 0.00194% per month or 0.023% per year, is highly consistent with that estimated in white men in the US (0.03%) using conventional methods (Daniel and Debanne, 1997).

**Table 1:** **Derived Markov chain parameters, based on Monte Carlo simulations of 600 pairs of case rates for infliximab- and etanercept-associated TB that span published values**.

**Parameter**	**Infliximab**	**Etanercept**	**Ratio (I:E)**
*R*	0.208 (0.195–0.237)	0.0158 (0.012–0.026)	12.1 (8.7–17.3)
*P*	1.0 (1.0–1.0)	1.0 (1.0–1.0)	1.0 (1.0–1.0)
*L*	0.0014 (0.00085–0.00228)		
*N*	0.0000194 (0.000013–0.000026)		

*R, monthly proportion of latent TB infections undergoing reactivation; P, proportion of new infections progressing directly to active TB; L, proportion with latent TB infection at baseline; N, monthly proportion acquiring new infection with Mycobacterium tuberculosis. Iterative nonlinear regression analysis was used to identify model parameters that fit the published time-to-onset distributions. Values are the median and interquartile range. From (Wallis, 2008)*.

**Figure 3:**
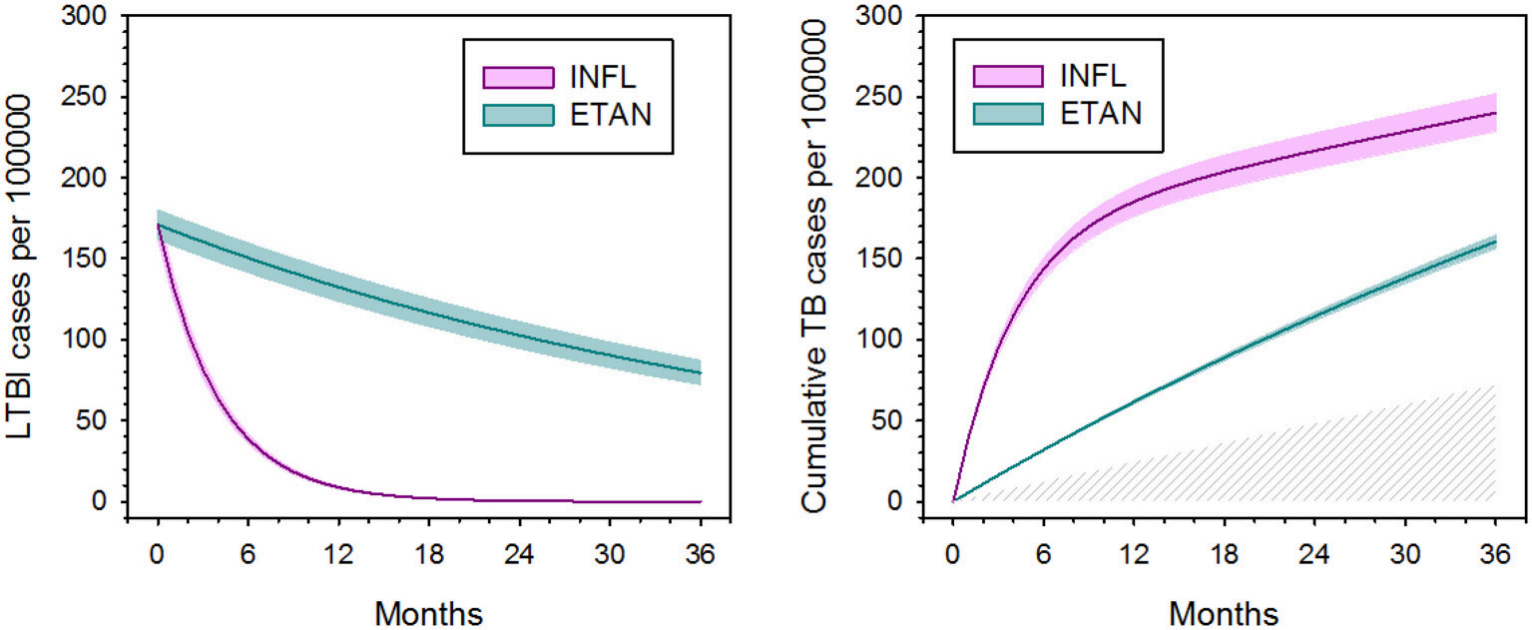
**Apparent effect of tumor necrosis factor blockade on latent tuberculosis infection (left) and total number of active tuberculosis cases (right), based on hidden Markov modeling and Monte Carlo simulation**. Colored shading indicate 95% confidence intervals. Latent TB infection is defined functionally by its capacity to reactivate. The hatched area (**right**) indicates TB cases arising due to new infection, which occur equally for both agents. Adapted from (Wallis, 2008), with permission.

Markov model parameters at the case rate ratio of 1.9 indicated by the FDA AERS data (Wallis et al., 2004a) were then used to examine 100,000 hypothetical patients over time (Figure 3). Infliximab treatment efficiently converted latent infections to active disease, leaving only 6% of latent infections remaining at 1 year. After that time point, the progression of new infection became the predominant cause of TB for infliximab-treated patients. In contrast, the low monthly rate of reactivation by etanercept resulted in reduced total numbers of cases, for which the contributions of reactivation and progression were nearly equal. After 2 years, infliximab had reactivated 3.4 times more cases than etanercept.

In 2013, Agliari et al. (2013) used formal stochastic modeling to confirm that the reduced tuberculosis risk after the first year of infliximab treatment was due to depletion of the pool of LTBI cases. The authors extended their analysis to examine the etiology of non-tuberculous mycobacterial (NTM) infections, making use of time-to-onset data for 239 anti-TNF treated patients (Winthrop et al., 2009). Unlike the case for tuberculosis, NTM infections continued to increase over time for both infliximab and etanercept, with no evidence that either agent depleted a pool of latent infections. Large numbers of NTM can be found in many environmental samples, including fresh water, aerosols, biofilms, and soils (Falkinham, 2002). As a result, repeated acquisition of NTM infection can easily occur during ordinary daily activities, leaving no evidence for reactivation of latent infection as a pathogenic mechanism.

Key findings from this modeling exercise are, that when LTBI is defined by its capacity to reactivate (as it was here): (1) the proportion of LTBI cases at baseline was strikingly low; (2) the proportion of tuberculosis cases arising from progression of new infection was surprisingly high; and (3) infliximab was markedly more efficient in reactivating LTBI than was etanercept.

## Predicting tuberculosis relapse

The identification of new regimens capable of shortening tuberculosis treatment without increasing the risk of recurrence has been a high priority for tuberculosis research for many years. However, the translation of the results of phase 2 trials into phase 3 trials has been a major challenge for the clinical development of such regimens. Phase 2 trials typically assess sputum culture conversion, whereas phase 3 trials assess relapse-free cure. Accordingly, regimen developers are keen to understand the quantitative link between these endpoints.

In 2013, a meta-regression analysis identified 2-month sputum culture status and treatment duration as independent predictors of recurrence, using data from 7793 patients treated with 58 diverse regimens of various durations published from 1973 to 1997 (Wallis et al., 2013). The selected regimens included all those in which 2-month culture results and relapses were reported, with one exception: If an early biomarker is to accurately predict clinical outcomes, treatment must continue as planned after the biomarker is measured. Rifampin is routinely administered for all of tuberculosis treatment, based on multiple studies finding reduced benefit in regimens in which it was prematurely discontinued (Jindani et al., 2004; Okwera et al., 2006). Regimens in which patients received rifampin only during the first 2 months were therefore excluded from the analysis, as in these cases, the drug's effects on culture conversion would be dissociated from those on relapse. Regimens were considered independently; a random intercept for study was included to account for differences among trials. Statistical methods in the analysis are summarized as follows.

Proportions were transformed using the logit function; those reported as zero were assigned values of 0.005 (0.5%) to permit logit transformation. The model included fixed effects for logit month 2 culture positive rate and for natural logarithm of treatment duration. The within-study variance of each study arm was fixed using the asymptotic variance of the logit-transformed recurrence proportion [1/Np(1-p)], where N was the arm's sample size and p was the recurrence proportion). The between-study variance was estimated by restricted maximum likelihood using the SAS MIXED procedure (Institute, 2008). Regression parameters were estimated via weighted least squares using the inverse of the sum of the within-study variances as the weight. From the fitted model, we predicted recurrence proportions at given proportions of month 2 culture positivity and treatment duration. Two-tailed 80% confidence intervals (CI) were calculated, as well as corresponding prediction intervals (PI) for a hypothetical trial with 680 subjects per arm. The upper limit of this interval thus identifies the recurrence rate with only a 10% chance of being exceeded in a typical phase 3 trial (i.e., 90% power). The 10% value had been selected as the highest risk of failure likely to be considered acceptable by a pharma sponsor during the planning of such a trial. The prediction error variance on the logit scale was *SE*^2^ + *Vs* + *1/N*_*new*_*q* (1−*q*), where *q* was the model-predicted logit recurrence proportion at a given level of month 2 culture positive rate and treatment duration, *SE* was the standard error of *q, N*_*new*_ was the number of subjects per arm of the hypothetical trial, and *Vs* was the estimated variance associated with the study. The intervals were formed on the logit scale and back-transformed to an ordinary scale. SAS code for the model is available on request (Wallis et al., 2013).

**Figure 4:**
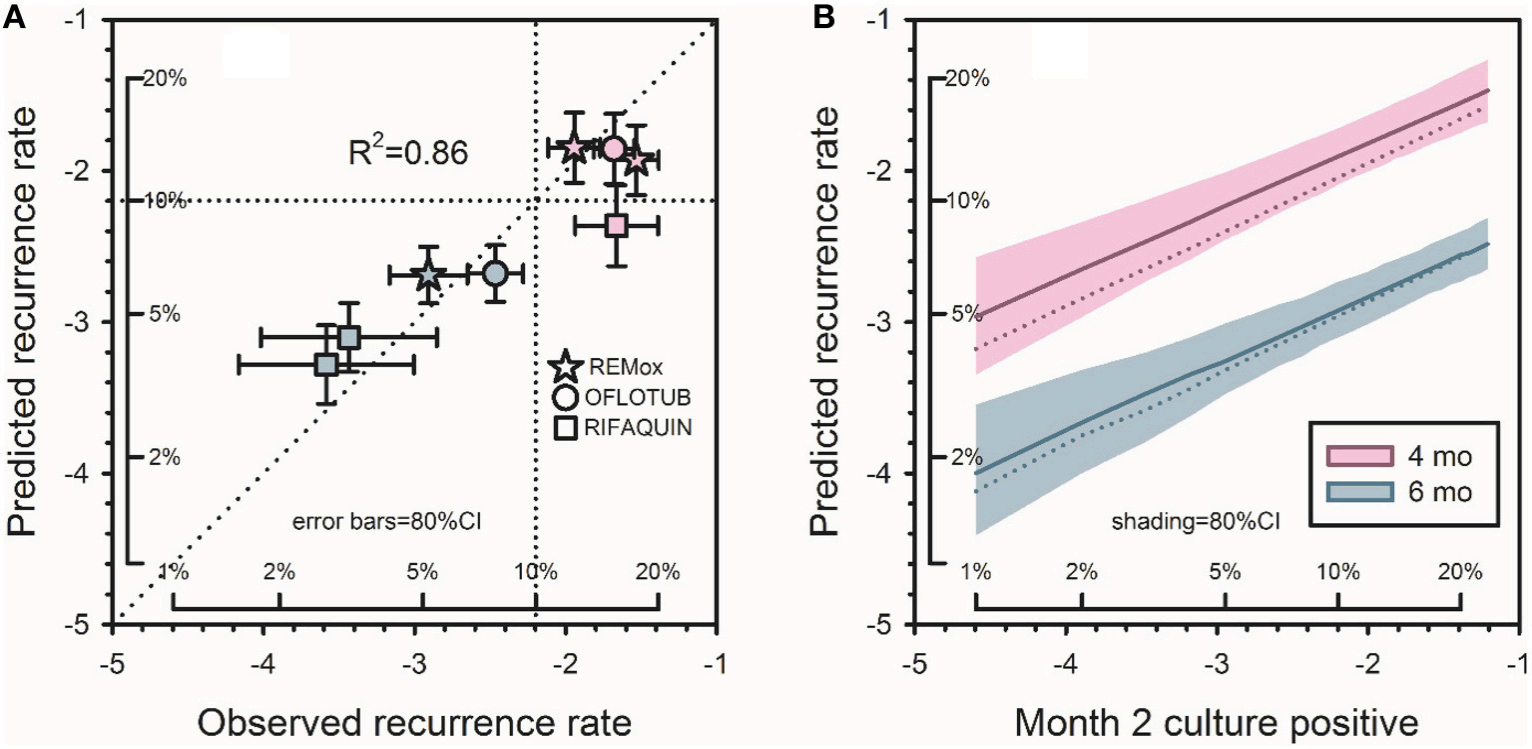
**Predicting tuberculosis recurrence based on month-2 culture**. **(A)** Observed recurrence rates for REMox, RIFAQUIN, and OFLOTUB, in relation to predicted rates based on data from studies published from 1973 to 1997. Axes in this figure indicate logit-transformed recurrence risk, with insets indicating corresponding numeric proportions. Red symbols indicate 4 month regimens; blue symbols indicate 6 month regimens. **(B)** Predicted recurrence for regimens of 4 and 6 months duration. Solid lines indicate updated predictions including the 3 recent trials; the dotted lines, the original predictions. Shading indicates the confidence interval for the revised predictions. The target month-2 positive rate for new 4-month regimens remained at 1%. Adapted from (Wallis et al., 2015), with permission.

The resulting model predicted that for a new 4-month regimen to reduce to 10% the risk of a relapse rate >10% in a typical phase 3 trial (*N* = 680/arm), it would reduce to 1% the proportion of culture positive after 2 months of treatment. The 1% target was far lower than anticipated, and met considerable skepticism. At that time, 5 phase 2 trials of 6 regimens containing gatifloxacin or moxifloxacin had reported month-2 culture positive proportions of 8–29% (Burman et al., 2006; Rustomjee et al., 2008; Conde et al., 2009; Dorman et al., 2009; Wang et al., 2010). The 2013 model predicted that if administered for only 4 months, all 6 regimens would yield unsatisfactory recurrence rates (10.4–19.4%; Wallis et al., 2013). In 2014, 3 independent phase 3 trials (REMox, OFLOTUB, and RIFAQUIN) published results for four unsuccessful fluoroquinolone-containing 4-month regimens (Gillespie et al., 2014; Jindani et al., 2014; Merle et al., 2014). The relapse rates of these regimens (12.5–17.8%) were highly consistent with those predicted based on 30 year-old data (10.4–19.4%). In 2015, the model was validated by analyzing the relapse rates across all arms in the three recent studies according to within-study month-2 culture data (Wallis et al., 2015). Predicted and observed rates were highly correlated (*R*^2^ = 0.86, Figure 4A). Updating the model to include data from all 66 regimens and 11181 patients (Table 2) had minimal effect on its predictions (Figure 4B).

**Table 2:** **Parameter estimates of original and revised linear meta-regression models**.

**Parameter**	**Estimate**	**SE (CV%)**	***P***
**ORIGINAL MODEL**			
Intercept	2.1471	0.6092 (28.4%)	0.0018
Natural log treatment duration	–2.2670	0.2958 (13.0%)	< 0.0001
Logit month 2 culture positive rate	0.4756	0.1063 (22.4%)	< 0.0001
**REVISED MODEL**			
Intercept	2.5289	0.4931 (19.9%)	< 0.0001
Natural log treatment duration	–2.5018	0.2299 (9.3%)	< 0.0001
Logit month 2 culture positive rate	0.4399	0.1004 (22.0%)	< 0.0001

*SE, standard error; CV, coefficient of variation. From (Wallis et al., 2015)*.

The 2015 publication described the accurate prediction of outcomes in a fourth study of 4-month treatment of tuberculosis in patients without cavitary disease at diagnosis and with negative cultures after 2 months of treatment (Johnson et al., 2009). Two additional small studies, of 12-month regimens for multi-drug resistant tuberculosis are also of interest in that both reported 2-month culture status and neither found relapses during the subsequent year. In the first, conducted in 100 patients in Cameroon (Kuaban et al., 2015), 13% were culture positive at month 2, yielding a predicted relapse rate of 1.1%. Poisson analysis indicates in a sample of 100 with a “true” rate of 1.1%, the most likely results are 0 events (33% likelihood) or 1 event (36% likelihood). In the second, conducted in 65 patients in Niger (Piubello et al., 2014), 6% were positive at month 2, yielding a predicted relapse rate of 0.7%. In this case, Poisson analysis indicates the most likely outcome would be 0 events (63% likelihood). Thus, the model accurately predicted the outcomes of both MDR-TB trials.

The finding that the model accurately predicts outcomes of contemporary studies despite significant differences in regimen composition, treatment duration, and geographic region indicates the model is robust and generalizable, thus meeting the criteria of Chau et al. as a “known valid” biomarker (Chau et al., 2008). An abbreviated version of the 2015 model appears as an online calculator at http://www.rswallis.com/Pages/TBrelapsecalculator.aspx.

## Summary

These 3 diverse examples illustrate how mathematic models can help advance our understanding of basic aspects of *Mtb* biology as they affect drug and vaccine development. In each case, analysis of modest data sets in what might be called “thought experiments” provided a remarkably clear picture of what are otherwise invisible stages of tuberculosis pathogenesis (latency and reactivation). In the case of TNF blockers, this reflected the particularly unique power of Markov modeling and Monte Carlo simulations when used in combination to reveal hidden events (Sonnenberg and Beck, 1993).

The third example, predicting the relapse risk of new tuberculosis regimens, reflects specific advances in the science of pharmacometrics over the past 2 decades. These were developed in the pharmaceutical industry to help avoid failures in phase 3 trials by identifying the factors necessary for success. The resulting techniques, including meta-dose-response modeling and meta-regression analysis, can help predict whether the effect observed on a biomarker in phase 2 will be sufficient to translate to a clinical outcome in phase 3. The root cause of the 3 unsuccessful fluoroquinolone trials appears to lie in a failure to ask this fundamental question. Mathematical models such as those described here will be important tools to guide the design of future tuberculosis clinical trials.

## Author contributions

The author confirms being the sole contributor of this work and approved it for publication.

### Conflict of interest statement

The author declares that the research was conducted in the absence of any commercial or financial relationships that could be construed as a potential conflict of interest.
